# The status of clinical trials: Cause for concern

**DOI:** 10.1186/1479-5876-8-65

**Published:** 2010-07-07

**Authors:** Robert B Nussenblatt, Curtis L Meinert

**Affiliations:** 1 Laboratory of Immunology, National Eye Institute, National Institutes of Health, Building 10, Room 10N112, 10 Center Drive, Bethesda, MD 20892 USA; 2 Johns Hopkins Center for Clinical Trials, 615 N. Wolfe Street, Baltimore, MD 21205 USA

## Abstract

**Background:**

Americans see clinical research as important, with over 15 million American residents participating in NIH-sponsored studies in 2008 and growing yearly.

**Methods:**

Documents reporting NIH supported Clinical Research projects were reviewed.

**Results:**

When compared with other studies, the number of interventional Phase III and Phase IV trials have decreased from 20% to 4.4% from 1994-2008.

**Conclusions:**

This finding most likely has occurred for several reasons. One reason is that the physician lacks an infrastructure for designing and carrying out trials. This lack is because of an absence of a coordinated effort to train clinical trialists. It is clear that the Nation needs a more purposeful approach to developing and maintaining the infrastructure for designing and conducting clinical trials. Building it de novo trial by trial is profoundly inefficient, to say nothing about time consuming and error prone.

## Introduction

**Figure 1:**
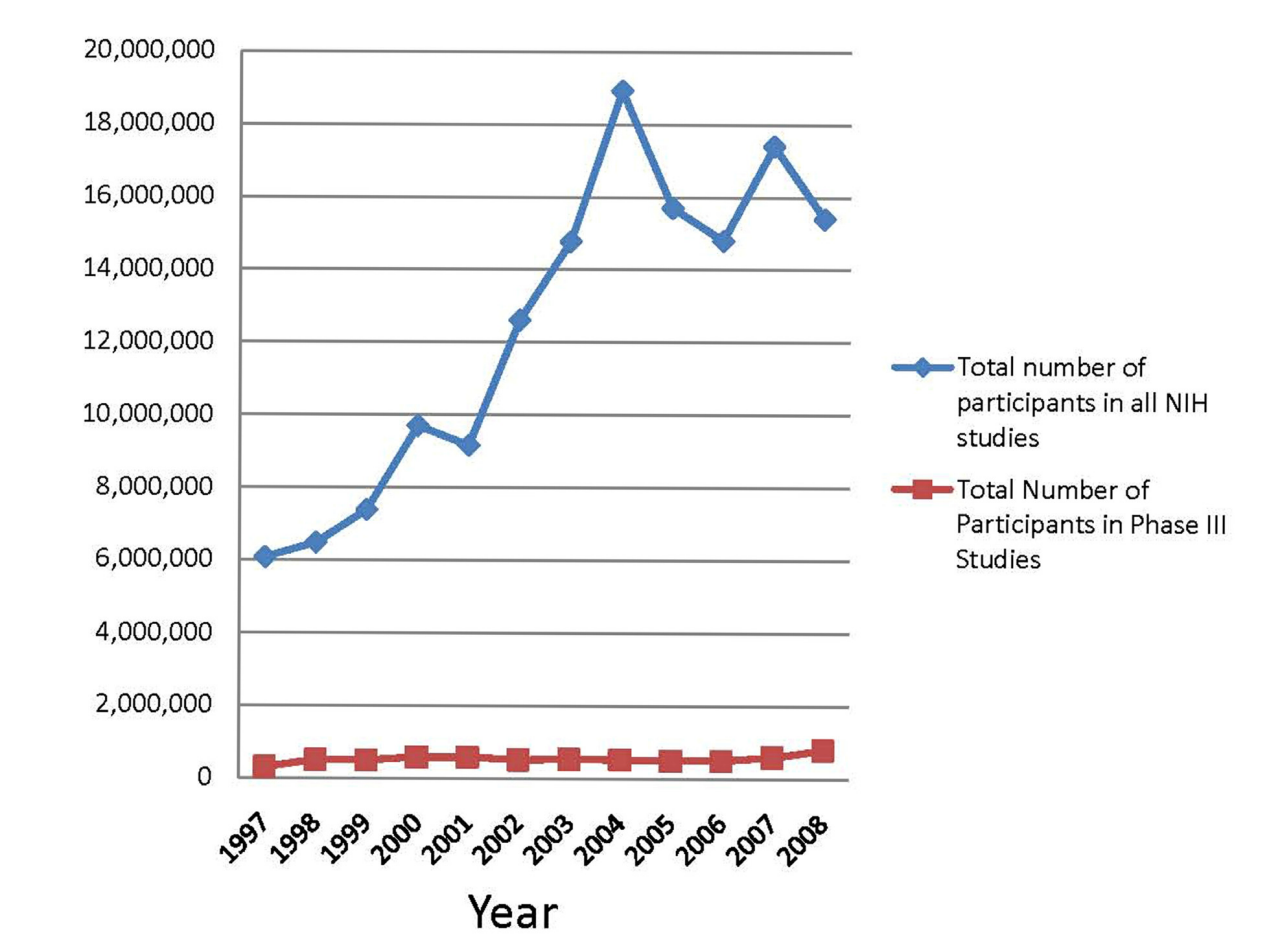
**Total number of NIH sponsored clinical studies over a ten year period**. The data were collected from yearly reports published by the NIH Office of Research on Women's Health, "Comprehensive Reports". http://orwh.od.nih.gov/inclusion/inclreports.html Accessed February 2010.

**Figure 2:**
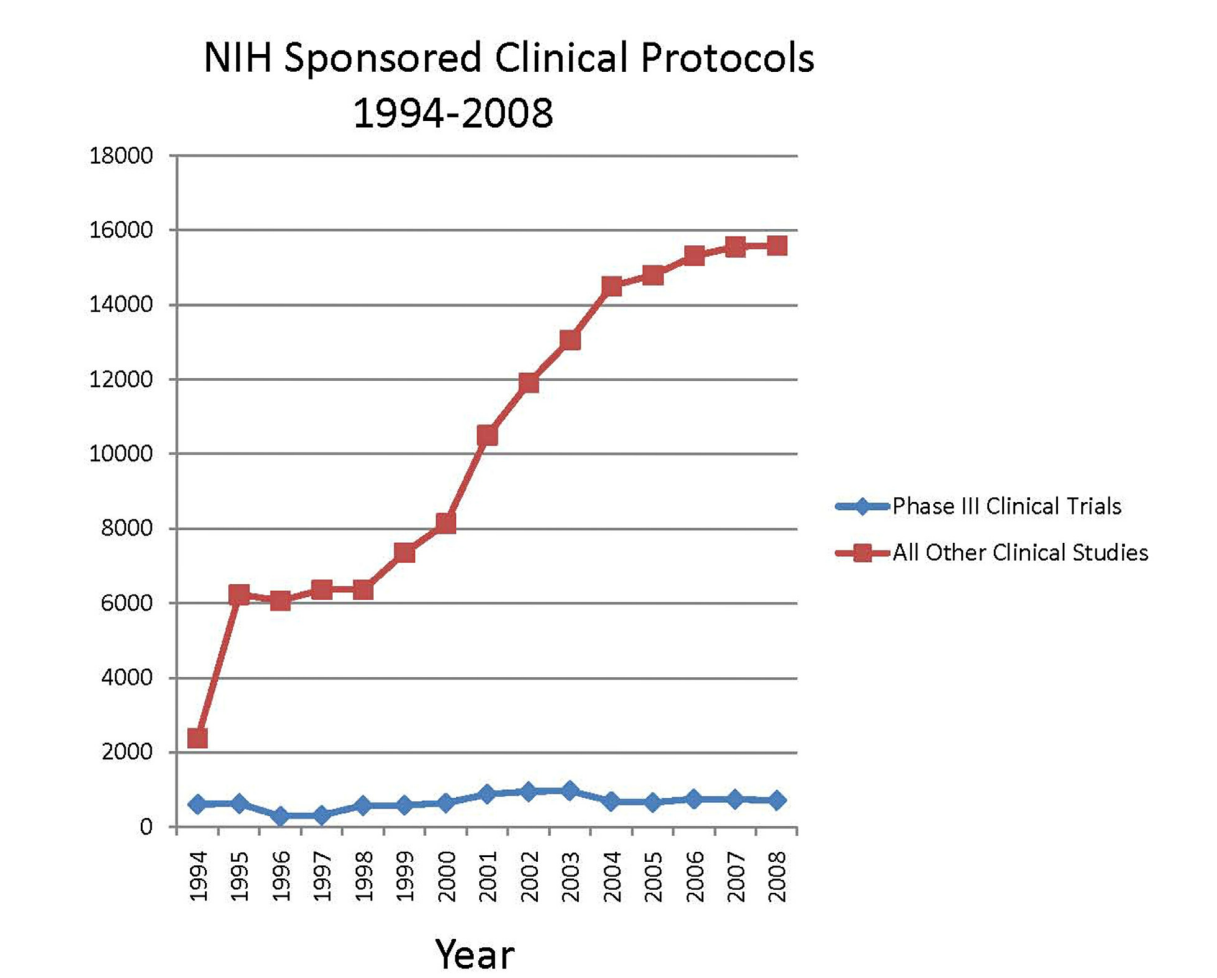
**Total number of patients in NIH sponsored clinical trials**. The data were collated from the same source as in Figure 1.

Scientific advances have been extraordinary in the last 50 years. We have seen areas of basic scientific research burgeon with the hope that they will soon translate into newer and more effective ways to treat or prevent human disease. As an offshoot of these advances new therapeutic agents, procedures and devices have appeared. The pharmaceutical industry has experienced decades of growth and success. It has been estimated that in 2004 over 30 billion dollars was spent on research and development in the United States by members of the Pharmaceutical Research and Manufacturers of America [1] It is clear that the American public sees the development of new treatments as important. We have seen a steady increase in participation by residents of the United States in clinical studies. For example, we know that over 15 million American residents participated in NIH-sponsored studies in 2008 [2], equivalent to almost 5% of the United States population. However, a closer look at this picture is not so reassuring. While clearly one can point to impressive advances therapeutically, it can be argued that the Nation's public health goals are not being met nor are important scientific agendas advancing. Over the past decade, the total number of NIH-supported clinical studies* of all types [3] has increased dramatically (Figure 1). There was a striking increase from 1999 to 2008, no doubt a consequence of an almost doubling of the NIH budget that occurred over those years. However, what types of studies have been performed? Some of this is difficult to ascertain, but one can look at NIH-sponsored Phase III studies** [4] and for which there are data. Unlike the increase in the overall increase in studies involving human subjects, the number of Phase III clinical trials has decreased. The percentage of Phase III trials relative to all clinical studies has decreased from 20% in 1994 to 4.4% in 2008, and has been at this level for the past 5 years. In spite of the increase in participants in NIH studies overall, the number participating in Phase III trials has remained much the same since 1998 (Figure 2).

## Discussion

Another area of concern is the endangered physician/researcher. Academia is the training ground for future young investigators and/or the incubator for new diagnostic and therapeutic interventions. The physician/scientist is seen as playing a major role in the regeneration process. With the growing demands of clinical medicine and research we have seen that the mix of the two becomes more difficult to attain in academia. Recognizing the possible demise of such persons in the NIH intramural program, people at the NIH are trying to foster a revival. Recently, many intimately involved with clinical research have voiced concern that the physician/scientist is in danger of disappearing in the academic world and away from the NIH as well [5,6]. Investigator-initiated clinical research is mainly NIH-sponsored in the United States. Studies generated by physician/scientists represent the life blood of the clinical research system in the United States.

While industry-sponsored studies are frequently well-designed, Friedberg et al [7] reported that pharmaceutical industry oncology drug company-sponsored trials were less likely to report unfavorable qualitative conclusions than those studies sponsored by non-profit organizations (5% vs. 38%, p = 0.04). Marcia Angell has written extensively about the "Me-too" drugs of the Pharmaceutical Industry. Angell [8] reported that from 1998 to 2003, 487 drugs were approved by the FDA [9], and of those 379 (78%) were classified by the FDA as having similar qualities to those already on the market. Further, 68% of these were new formulations or combinations of old drugs. Only 67 (14%) were considered to be new formulations.

An additional confounding issue is "globalization" of drug development and testing. Clinical research is an area that has been dominated by the United States and is an important financial segment of the "new technologies" portion of our economy. One FDA report [10] dealing with the tracking of off-shore clinical trials was telling. It reported that the number of foreign clinical investigators conducting clinical research under an IND increased 16-fold from 1990 (271) to 1999 (4,458). In addition the number of countries in which FDA tracked drug studies increased from 28 in 1990 to 79 in 1999 [10].

In addition to a diminishing supply of clinical researchers, we would suggest that a major difficulty lies with the lack of a coordinated/focused effort to train persons in the design, conduct, or analysis of trials. As a result, most academic institutions lack people to work with the clinician investigator. Almost any clinical investigator in any university system knows that if he/she wishes to initiate a trial with any significant number of participants, industry partners are needed. The lack of infrastructure is due in part to the failure to recognize need for it until it is too late. All too often there is the perception that it is not needed. Even if there is such recognition it is difficult to achieve because the people required are scattered throughout Universities, largely isolated one from another. The production of trialists is low. For example, over the period 1991-2002, of 232 PhD dissertations at the Bloomberg School of Public Health of the Johns Hopkins University, 10 were devoted to clinical trial topics; at the University of Washington during a similar period, 3 of 132 and at Tulane University's School of Public Health there were no clinical trial subject dissertations amongst 52 completed [11]. It is clear that the leadership of the NIH is aware of the problems, as signaled by the previous director (Elias Zerhouni) and his "Roadmap" [12]. Dr. Zerhouni wrote in 2005, "It is the responsibility of those of us involved in today's biomedical research enterprise to translate the remarkable scientific innovations we are witnessing into health gains for the nation" [13]. One may criticize specific points in the roadmap, but it offers an important vision and applauds it as a step forward. However, that said we note an absence of attention to creating and maintaining infrastructures for trials and training trialists.

One way to address the issue would be for the NIH intramural program to initiate an integrated program that leads to the goal of buttressing the clinical trials infrastructure. The leaders of the NIH intramural program have been discussing how best to re-define the intramural role. Revitalizing the clinical research effort could be one such area. This goal would support the programmatic needs of all of the NIH Institutes and Centers. With more than a thousand clinical research protocols active at any point in time, the intramural program could become a laboratory for clinical research, a place for exploration of more efficient and novel ways to do clinical research, and a place where all the important players, government, academia and industry, could collaborate. A core trans-Institute training program could be established on the NIH campus to train personnel involved at all levels of the clinical research infrastructure.

The running of clinical trials demands a concentrated effort by a team, including trialists, information technology specialists, and bioethicists, in addition to clinicians; training must include them all. In a trans-NIH effort, one could envisage a stronger working relationship with the Food and Drug Administration and industry. An NIH/FDA effort could evaluate new approaches to perform and record clinical research in order to enhance patient safety and streamline administrative approaches. To complete the paradigm shift one should envisage involvement of the pharmaceutical industry; advantages for those in industry that participate in these programs should be considered. Mechanisms now exist to permit private support for intramural programs. A second mechanism would the use of the Clinical and Translational Science Award (CTSA) mechanism [14]. As per the NIH Roadmap, the intent is to encourage novel approaches and methods to clinical and translational research. While presently seeking ways to improve clinical design, biostatistics, ethics, and informatics, the program should nurture the development of clinical trial teams, including training and mentoring of the trialist.

Those concerned with America's public health and the advancement of medical care and diagnostics must look to new paradigms to invigorate the clinical research process, and the intramural program and the CTSA could play an important new role in meeting the Nation's goal of improving the health of its citizens.

*NIH definition of Clinical Studies: "The definition of Clinical Studies for this study is taken from 45CFR46. Clinical research is defined as: 1. Patient oriented research. Research conducted with human subjects A (or on material of human origin such as tissues, specimens, and cognitive phenomena) for which an investigator (or colleague) directly interacts with human subjects. Excluded from this definition are in vitro studies that utilize human tissues that cannot be linked to a living individual. Patient-oriented research includes: (a) mechanisms of human disease, (b) therapeutic interventions, (c) clinical trials, and (d)development of new technologies, (2) Epidemiologic and behavioral studies, (3) Outcomes research and health services research [3].

**The NIH-defined Phase III Clinical Trial: " ... usually involving several hundred or more human subjects, for the purpose of evaluating an experimental intervention in comparison with a standard or controlled intervention or comparing two or more existing treatments. Often the aim of such investigation is to provide evidence leading to a scientific basis for consideration of a change in health policy or standard of care." [4]

## Competing interests

The authors declare that they have no competing interests.

## Authors' contributions

Both authors conceived of the study, and participated in its design and coordination and helped to draft the manuscript. Both authors read and approved the final manuscript.
